# Consumption of fortified infant foods reduces dietary diversity but has a positive effect on subsequent growth in infants from Sumedang district, Indonesia

**DOI:** 10.1371/journal.pone.0175952

**Published:** 2017-04-20

**Authors:** Aly Diana, Simonette R. Mallard, Jillian J. Haszard, Dwi Monik Purnamasari, Ikrimah Nurulazmi, Pratami D. Herliani, Gaga I. Nugraha, Rosalind S. Gibson, Lisa Houghton

**Affiliations:** 1 Faculty of Medicine, Universitas Padjadjaran, West Java, Indonesia; 2 Department of Human Nutrition, University of Otago, Otago, New Zealand; Institut de recherche pour le developpement, FRANCE

## Abstract

Stunting and underweight among under-five children in Indonesia are common, raising public health concerns. Whether inappropriate complementary feeding (CF) practices compromise optimal growth during late infancy in Indonesia is uncertain. Therefore we characterized and evaluated CF practices in Indonesian infants and investigated their relationship with subsequent growth. We enrolled breastfed infants at 6 months of age (n = 230); and followed them at 9 (n = 202) and 12 months of age (n = 190). We collected socio-demographic and anthropometric data and two-day in-home weighed food records. Relations between WHO CF indicators, sentinel foods, and energy and micronutrient intakes at 9 months and growth at 12 months were explored using multiple linear regression. Stunting and underweight increased from 15.8% and 4.4% at 6 months to 22.6% and 10.5% at 12 months, respectively. Median intakes of calcium, iron, zinc, and riboflavin were below WHO recommendations. Infants consuming fortified infant foods (FIFs) at 9 months had diets with a lower dietary diversity (DD) score (2.3 vs.3.0), energy density, median energy (250 vs. 310 kcal/d) and protein (6.5 vs. 9.1 g/d) intake than non-consumers (p<0.01), despite higher intakes of calcium, iron, and vitamins A and C (p<0.001). Positive relations existed for 9-month consumption of iron-rich/iron fortified infant foods with length-for-age Z-score (LAZ) at 12 months (β = 0.22; 95% CI: 0.01, 0.44; P = 0.04), and for fortified infant foods alone with both LAZ (β = 0.29; 95% CI: 0.09, 0.48; P = 0.04) and weight-for-age Z-score (β = 0.14; 95% CI: 0.02, 0.26; P = 0.02) at 12 months. The positive association of FIFs with subsequent growth may be attributed to their content of both powdered cow’s milk and multi-micronutrient fortificants. Nonetheless, mothers should not be encouraged to over-rely on FIFs as they reduce DD.

## Introduction

Childhood undernutrition remains a significant problem in Indonesia, even though there have been major improvements in health over past decades. In 2013, among under-five children, mortality rates were 32 per 1000 live births [1], 20% were underweight, and 37% were stunted [2]. Such growth faltering often begins around 4–7 months of age [3, 4], and is accompanied by a high prevalence of anemia and co-existing micronutrient deficiencies [5, 6].

Despite very high national breast feeding rates at six months (76%) [7], progress in improving complementary feeding practices in Indonesia has been slow [8]. The rice-based gruels traditionally used for complementary feeding often have low energy and nutrient density [9]leading to nutrient deficits when compared with estimated needs [10], particularly for calcium, iron, and zinc [11]. Such deficits may be exacerbated both by the displacement of breast milk if complementary feeding is introduced prior to six months of age, and poor appetite induced by infection [12].

The World Health Organization (WHO) has set a global target to reduce childhood stunting by 40% by 2025 [13]. Achieving this goal is a priority for Indonesia. Guiding principles for the complementary feeding of the breastfed child have been developed [14] but whether they are widely practiced in Indonesia is uncertain. Therefore, in an initial survey, we studied a group of breastfed infants from the Sumedang district, West Java province, Indonesia, a district with rates of stunting (41.1%) and underweight (14.6%) comparable to the national prevalence in Indonesia [2]. Our objectives were to: 1) characterize and evaluate prevailing complementary feeding practices, 2) assess the adequacy of energy and nutrient intakes during complementary feeding, and 3) investigate relationships between WHO complementary feeding indicators, local sentinel food groups, nutrient adequacy, and subsequent infant growth in a cohort of breast-fed infants at 6, 9, and 12 months of age. In view of the increasing use of fortified infant foods by mothers in Asia [15, 16], we also examined these relationships with the consumption of fortified infant foods. We envisaged that our study might lead to the development of interventions to improve complementary feeding practices and reduce risk of infection. Appropriate feeding practices during early childhood are essential because interventions after the first two years appear to have little impact on subsequent child growth [17].

## Methods

### Study site and participant recruitment

This prospective cohort study was conducted in Tanjungsari, Sukasari, and Pamulihan sub-districts of Sumedang district, West Java, Indonesia between August 2014 and August 2015. Sumedang district has a population of 1.1 million and an area of ~152 hectares, of which 22% is used for rice paddy plantation. The climate is tropical with rain most months and only a short dry season [7]. The majority of the population are Muslim of Sundanese ethnicity. Infants were enrolled at 6 months of age and randomly selected from all the villages (n = 30) in the three sub-districts using local birth registry data. Selected measurements were recorded on the infants (mean,SD) at 6 (±4 weeks), 9 (±4 weeks), and 12 (±4 weeks) months of age.

Infants were eligible for inclusion if they were not premature (>37 weeks gestation), ≥1500 g at birth, apparently healthy with no evidence of chronic disease or acute malnutrition, and had received predominant/exclusive breastfeeding until 6 months of age. The sample size of 200 healthy breastfed infants was calculated to be able to estimate the prevalence of stunting (length-for-age Z-score (LAZ) < -2SD) with a 95% confidence interval precision of at most 7%. This sample size also allows us to conduct regression analyses involving several variables with at least a minimum of 10 respondents per variable in the model [18]. Ethical approval was obtained from the Human Ethics Committees of Padjadjaran University, Indonesia, and the University of Otago, New Zealand. Informed written consent to participate in the study was given by the parents or primary guardians of the infants. Participants were free to withdraw from the study at any time.

### Socio-demographic and health status

Pre-tested questionnaires were administered by trained research assistants during home visits when data on socio-demographic collected at baseline, and health status, sanitation and hygiene at 6, 9 and 12 months of age. The measures of health status collected included morbidity in the last two weeks arising from fever, cough, diarrhea, vomiting, history of hospitalisation, and immunisation status. Sensitive information on sanitation facilities was recorded by in-home observations.

### Anthropometry

Weight and recumbent length were measured on infants at aged 6, 9, and 12 months by trained research assistants with infants unclothed or wearing a weighed diaper using standardized techniques [19] and calibrated equipment [20]. Weight was recorded to the nearest 10 g (Seca 334, Seca GmbH & Co. KG., Hamburg, Germany) and length to the nearest millimeter (Seca 417, Seca GmbH & Co. KG., Hamburg, Germany). Measurements were recorded in duplicate, or triplicate if the difference between first and second measurement was more than recommended [21]. Both inter- and intra-examiner technical error for each anthropometric measurement were acceptable based on 20 (non-study) 6-month old infants. Maternal height (to nearest millimeter) and weight (to nearest 100 g) at recruitment were also measured at baseline. Z-scores for length-for-age, weight-for-length (WLZ), and weight-for-age (WAZ) were calculated using the most recent WHO growth reference data [22] and WHO AnthroPlus 3.2.2. None of the infants had unacceptably extreme anthropometric values defined as ± 3 SD [23].

### Assessment of complementary food intakes

Trained volunteer community health workers (cadres) weighed (Kitchen Scale EK3131, Camry Electronic Ltd, Guangdong, China) 12-h day-time food intakes of infants in their homes on two non-consecutive days within one week. Mothers recorded the night-time foods and amounts consumed during the previous 12-h in household measures, with amounts converted to gram equivalents by the community health workers (cadres) which were used to estimate total 24-h intakes of complementary foods at 6, 9, and 12 months of age. Ingredients of mixed dishes and the amount consumed were also weighed and the weight of the actual ingredients consumed by the child calculated [24]. For purchased foods, average recipes were compiled from local recipes and used to calculate the average energy and nutrient content of each purchased food.

### Compilation of a local complementary food composition table

Energy and selected nutrient composition values for the complementary foods consumed were taken primarily from the Indonesian food composition table developed for the SMILING Project [25] and the FAO/INFOODS Food Composition Databases for Asia [26], augmented where necessary with nutrient values from the US Department of Agriculture [27] and ProPan 2.0 [28]. Micronutrients chosen were based on the availability of reliable nutrient composition data of Indonesian foods and their perceived public health relevance for complementary feeding. Nutrient values for all wheat flour products were adjusted to reflect the government mandatory fortification levels (thiamine 2.5 mg/kg, riboflavin 4 mg/kg, iron 50 mg/kg, and zinc 30 mg/kg) [29]. For 13 frequently consumed commercially processed plant-based complementary foods, values for iron, zinc, calcium were compiled from chemical analysis of at least three samples of each item, as described earlier [30, 31]. Vitamin A values were re-calculated where necessary and presented as retinol activity equivalents (RAE) [32]. Nutrient values for 54 mixed dishes were calculated from weighed recipe data taking into account appropriate yield and retention factors [33]. Phytate values were compiled from the literature [31, 34, 35] or imputed where necessary, and adjusted for differences in moisture content. This local food composition table was then used with the digitized 24-h dietary records to calculate the energy and nutrient intakes of the infants on each of the two-recorded days.

### Assessment of energy and nutrient intakes and adequacy of complementary foods

Median (25^th^, 75^th^ percentile) daily intakes from complementary foods of energy and selected nutrients at 6, 9, and 12 months were calculated and compared with estimated needs from complementary foods alone based on FAO/WHO/UNU (2004) [36], energy requirements (per day; per kg/body weight), and WHO/FAO [37], and recommended nutrient intakes (assuming average breast milk volume and composition) [38]. For zinc, the recommended nutrient intake compiled by Brown et al. [39] was used. Median (25^th^, 75^th^ percentile) daily intakes from complementary foods of energy and selected nutrients at 9 months were also calculated for those infants who did or did not consume fortified infant products, primarily to assess the impact of fortification of infant foods on the complementary feeding regime. A summary indicator—overall mean adequacy ratio (MAR)—was also calculated to reflect the overall adequacy of the micronutrient intakes of the infants at each age. The MARs are derived from nutrient adequacy ratios (NARs) by dividing the actual nutrient intakes from complementary foods for each infant by the corresponding estimated needs, expressed as percentages. After capping the NARs at 100%, they were then averaged to generate MARs for all the infants, and for those who did or did not consume fortified infant foods at 9 months [40]. Median (25^th^, 75^th^ percentile) micronutrient densities (amount per 100 kcal food) were also calculated for the infants at each age and compared with the desired nutrient densities [10].

### Compilation of infant and young child (IYCF) complementary feeding indicators

All infants were breast-fed, with the exception of four infants at 12 months of age. The individual weighed food record data was used to assess whether each infant met or did not meet (Yes/No) the five core WHO population-level complementary feeding indicators [41, 42], and the proportion of the infants at aged 6, 9, and 12 months meeting each indicator was then calculated. The WHO indicators used were: 1) consumption of solid, semi-solid or soft foods; 2) minimum dietary diversity (MDD) ≥4 food groups; 3) minimum meal frequency (MMF); 4) consumption of a minimum acceptable diet (MAD); and 5) consumption of iron-rich (i.e., flesh foods) and/or iron fortified foods. For the MDD indicator, the seven WHO food groups were used [41] with no minimum quantity of consumption defined: 1) grains, roots, and tubers; 2) legumes and nuts; 3) dairy products (milk, yogurt, cheese; infant formula); 4) eggs; 5) flesh foods (meat, fish, poultry, and liver/organ meats); 6) vitamin A–rich fruits and vegetables; and 7) other fruits and vegetables. Significant ingredients in a mixed dish were allocated to food groups separately. In addition, the proportion of infants at each age consuming four WHO nutrient-dense sentinel food group indicators (flesh foods, dairy products, eggs, and animal-source foods), and a local sentinel food termed “fortified infant foods” was also calculated. The fortified infant foods comprised infant formulae, fortified infant cereals and rusks, many of which also contained significant amounts of dried milk powder. Clear broths from simmered dishes and soups were not included in the compilation of the MDD, MMF or MAD indicators.

Dietary diversity was calculated by summing the number of the seven food groups [41, 42] consumed at least once during the two days of food records at each age. MDD was defined as the consumption of four or more food groups at least once during the two record days. Meal frequency was calculated as an average of the sum of the number of meals (other than trivial amounts <10 g) consumed over each of the two food record days. MMF for breast-fed infants was defined as having consumed 2 or more solid, semisolid, or soft meals per day for each of the two food record days at 6 months and 3 or more meals per day at 9 and 12 months of age [41]. MAD was defined as meeting the requirements for both MDD and MMF for breast-fed infants at each age. Consumption of iron-rich/iron-fortified foods was defined as having consumed a flesh food and/or an iron-fortified food specially designed for infants and young children at least once out of the two record days. We also calculated the data for the WHO IYCF indicators and the four sentinel food groups and fortified infant foods based on day 1 only in order to compare our one day data with previously published data based on single 24-hr recalls.

### Statistical analysis

Data were transferred into Stata^®^ 12 (StataCorp LP, Texas, USA), and descriptive and comparative statistics calculated. All continuous variables were visually assessed for normality. Maternal education was categorized as primary school or less, secondary school, and college/university. An asset-based wealth index was calculated using principal component analysis based on asset variables recommended for use in the Indonesian Demographic Health Survey [1], and following the DHS Wealth Index guidelines [43]. This continuous index was then divided into quintiles from the lowest to highest household wealth. Spearman rank correlations were conducted to assess the relation between dietary diversity scores and NARs for all infants and stratified for those consuming or not consuming fortified infant foods at 9 months. Differences in the median intakes of energy and nutrients for those infants consuming and not consuming fortified infant foods at 9 months were tested using the Wilcoxon rank sum test. Differences between maternal/household characteristics of those infants consuming and not consuming fortified infant foods were tested using t-test (for continuous variables) and chi-squared (for categorical variables).

Multiple linear regression was used to investigate associations between each of LAZ, WAZ, and WLZ at 12 months as the dependent variables and, in turn, the following independent categorical variables at 9 mo: four core WHO IYCF indicators (MDD based on WHO seven food groups) [44], MMF, MAD, consumption of iron-rich/iron-fortified foods; consumption of the five sentinel foods groups: dairy products (FG 3); flesh foods (FG 4); eggs (FG 5); animal-source foods (dairy products, flesh foods and eggs), and fortified infant foods; and intakes of critical growth-limiting nutrients–protein, zinc, iron, calcium, and riboflavin (per day; per 100 kcal; per kg body weight).

All indicators were calculated from data generated over two non-consecutive days, with consumption of sentinel food groups counted when consumed at least once over the two days. All the fully adjusted linear regression analyses for assessing the impact of the complementary feeding regime on growth at 12 months included the following covariates: maternal height, sex, wealth index (quintiles), and maternal education as well as the respective 9-mo LAZ, WLZ, or WAZ data. Model assumptions were checked using residual plots, and Levene’s test was additionally used to assess homogeneity of variance and heteroscedasticity. All analyses were conducted using Stata^®^ 12, and a 2-sided p < 0.05 level of significance was used in all cases.

## Results

### Maternal socio-demographic status and infant characteristics at birth

**Fig 1:**
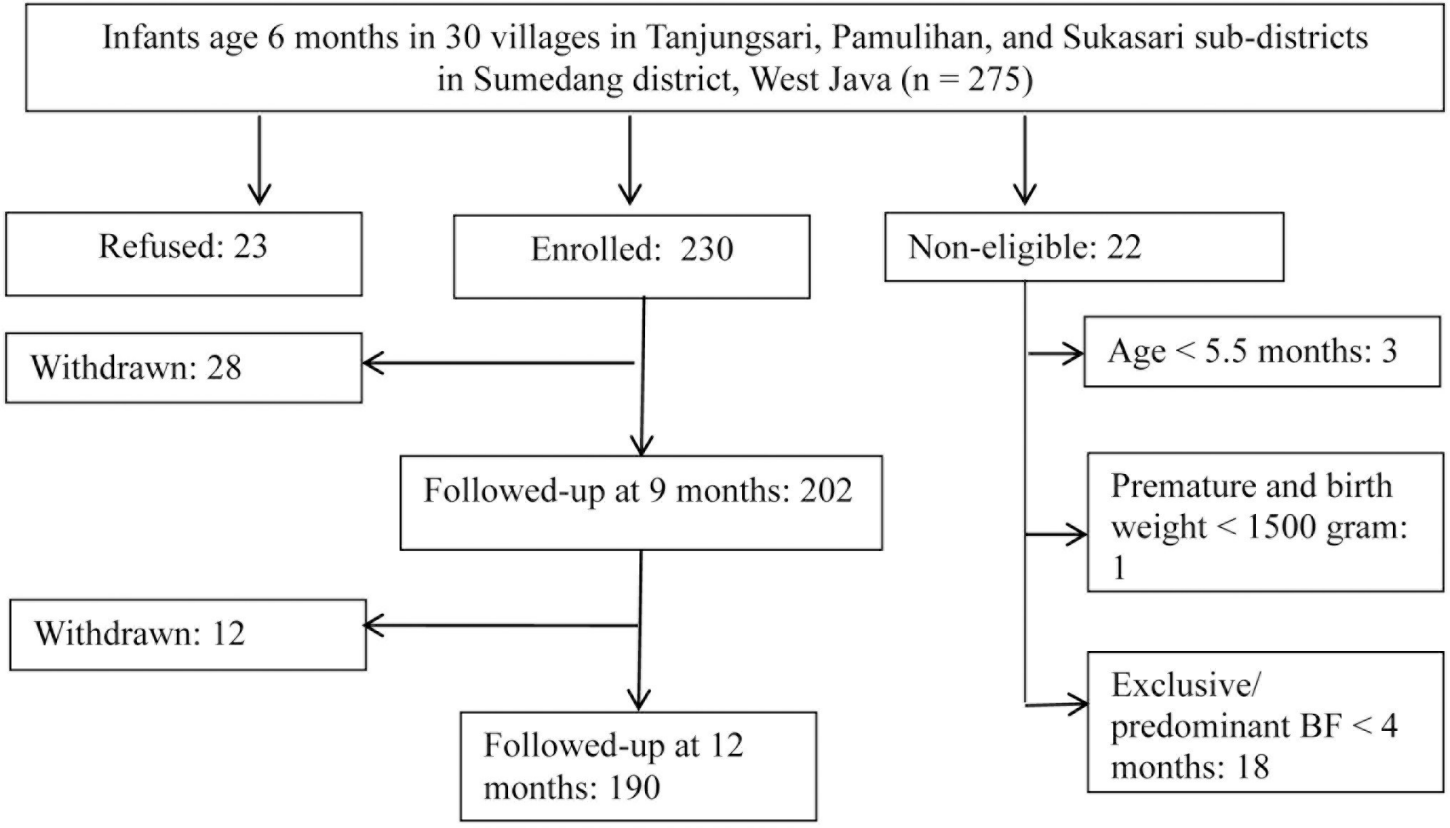
Flow of respondents in the longitudinal study.

A total of 275 6-month-old infants were approached initially, and details of the enrolment, refusal and subsequent withdrawals are shown in Fig 1. Of the 230 enrolled, 190 completed the study (completion rate, 82.6%); the main reason for withdrawal was the repeated blood sampling (Fig 1). Of the mothers, very few were underweight (7.3%), but 36.4% had body mass indexes (BMIs) classified as overweight or obese (i.e., BMI ≥25). Almost all mothers were housewives (91.6%), of whom more than 60% had completed at least secondary school; most fathers were manual labourers or farmers without a regular income (Table 1). Most households had an improved water source and toilet facility. Almost all the infants (96.8%) recruited had a birth weight between 2500–4500 g, as measured within 1–3 days by the midwives.

**Table 1:** Maternal, socio-demographic status and infant birth characteristics at baseline.

*Maternal characteristics*	n	
Age, y	226	27.5 ± 7.2
Height, cm	225	150.2 ± 5.2
Height <145 cm, %	35/226	15.5%
Body Mass Index (kg/m^2^)	220	23.7 ± 4.0
Underweight	16/220	7.3%
Normal	124/220	56.4%
Overweight	67/220	30.5%
Obese	13/220	5.9%
Education, %		
Primary school or less	93/229	40.6%
Secondary school	122/229	53.3%
College/university	14/229	6.1%
Number of pregnancies, %		
1	89/229	38.9%
2	82/229	35.8%
3 or more	58/229	25.3%
*Household characteristics*		
Father's education		
Primary school or less	109/229	46.7%
Secondary school	108/229	47.2%
College/university	12/229	5.2%
Father's occupation		
Regular wage earner	22/228	9.6%
Business or trade owner	69/228	30.3%
Manual labour	100/228	43.9%
Farmer	26/228	11.4%
Unemployed	11/228	4.8%
Improved drinking-water source	181/228	79.4%
Improved toilet facility	193/228	84.7%
*Infant characteristics*		
Female	123/230	53.5%
Birth weight, 2500–4500 g	211/218	96.8%
Diarrhea in the last 2 weeks*	20/183	10.9%
Complete vaccination**	165/185	89.2%

* At 9 months

** At 12 months: at least 1 BCG, 1 Polio, 1 DPT, 1 Hepatitis B, and 1 Measles (taken from immunisation card).

### Anthropometric status at 6, 9, and 12 months

Mean Z-scores (LAZ, WLZ, and WAZ) declined with increasing age (Table 2). The prevalence of stunting and underweight at 6 months increased from 15.7% (95% CI, 11.3–20.9%) and 3.9% (95% CI, 2.1–7.5%) respectively, to 22.6% (95% CI, 17.6–29.8%) and 10.5% (95% CI, 7.0–16.1%) at 12 months. In contrast, the prevalence of wasting was much lower (1.7%, 95% CI, 0.4–4.1% at 6 months) and relatively stable (3.2%, 95% CI, 1.4–7.0% at 12 months).

**Table 2:** Anthropometric characteristics of infants at 6, 9, and 12 months.

	6 mo	9 mo	12 mo
LAZ (mean, SD)*	-1.02 (0.97)	-1.16 (0.97)	-1.26 (1.00)
Prevalence of LAZ <-2 SD	36/230 (15.7%)	39/202 (19.3%)	43/190 (22.6%)
WLZ (mean, SD)*	0.45 (1.03)	0.17 (1.03)	-0.08 (1.08)
Prevalence of WLZ <-2 SD	4/230 (1.7%)	4/202 (2.0%)	6/190 (3.2%)
WAZ (mean, SD)*	-0.34 1.00)	-0.52 (1.02)	-0.66 (1.08)
Prevalence of WAZ <-2 SD	9/230 (3.9%)	11/202 (5.4%)	20/190 (10.5%)

LAZ (length-for-age Z-score), WLZ (weight-for-length Z-score), WAZ (weight-for-age Z-score), SD (standard deviation).

* p-value < 0.05.

### Infant and young child feeding practices

All the infants continued to be breastfed from six months of age, although by 12 months of age, four were no longer being breastfed. Almost all were receiving solid, semi-solid, or soft foods at 6 months, usually at least twice per day (Table 3). However, dietary diversity at 6 months was low, with less than 3% of infants achieving a MDD of ≥ 4 food groups, and thus few infants met the MAD threshold. Indeed, 63.6% of the infants were consuming only one or two food groups at 6 months, of which grains, roots and tubers, fruits and vegetables, and/or iron-fortified infant foods predominated (data not shown). At 9 and 12 months, the proportion consuming diets achieving the MDD and MAD thresholds increased markedly, as the range of food groups consumed rose (Table 3).

**Table 3:** Number and percentage of infants meeting five core WHO complementary feeding indicators and consuming four sentinel food groups at 6, 9, and 12 months.

WHO IYCF Indicator	6 mo n = 218	9 mo n = 193	12 mo n = 186
Introduction of solid, semi-solid, or soft foods	217 (99.5)	NA	NA
Minimum dietary diversity (≥ 4 food groups)	6 (2.8)	78 (40.4)	112 (60.2)
Minimum meal frequency	202 (92.7)	191 (99.0)	186 (100)
Minimum acceptable diet	6 (2.8)	78 (40.4)	112 (60.2)
Consumption of iron-rich/iron fortified infant foods	201 (92.2)	145 (75.1)	150 (80.1)
Sentinel food groups			
Dairy products	9 (4.1)	3 (1.6)	9 (4.8)
Flesh foods	5 (2.3)	105 (54.4)	140 (75.3)
Eggs	7 (3.2)	69 (35.8)	106 (57.0)
Animal-source foods	19 (8.7)	132 (68.4)	161 (86.6)
Fortified infant foods	200 (91.7)	69 (35.8)	23 (12.4)

Almost all of the infants at 6 months (92.2%) consumed iron-rich foods (i.e., iron-fortified foods or flesh foods), most of which were fortified infant cereal-based porridges and biscuits. Only nine children (i.e., 4.2%) consumed fortified formula milk. At age 9 and 12 months, even fewer infants consumed fortified formula milk (9 months: n = 2, 1.0%; 12 months: n = 8, 4.3%), with the proportion consuming cereal-based porridges and biscuits also declining progressively during this time. As a result, the portion size of fortified foods consumed (dry weight) averaged 12.1 g/portion (SD: 7.9) at 9 months, falling to an average of 7.8 g/portion (SD: 8.1) at 12 months. At 9 and 12 months, while consumption of fortified infant foods decreased progressively, consumption of flesh foods, notably meat balls and sausages, increased over this time period, providing a greater proportion of the iron-rich/iron fortified food group at 9 and 12 months (Table 3). It is noteworthy, that when the data in Table 3 were recalculated based on day 1 intakes only (S1 Table), then the proportion of infants meeting the MDD and MAD and consuming the four sentinel food groups and fortified infant foods was consistently lower at each age.

### Intakes of energy and selected nutrients and their micronutrient adequacy

Not surprisingly, median intakes from complementary foods of energy and most nutrients increased progressively with age (Table 4). Although median energy intakes at all ages were below the absolute estimated energy requirement, when expressed per kilogram body weight to account for the small size of the infants, median energy intakes met requirements at 9 and 12 months but not at 6 months. There were consistent deficits in several micronutrients, most notably for median intakes for calcium and iron at all three ages, riboflavin at 9 and 12 months, and zinc at 6 and 9 months of age (Table 4). For thiamine, no deficit was observed based on the assumption that the infants, irrespective of age, met their needs for thiamine from breast milk alone. Maternal thiamine status and thus breast milk thiamine concentrations were likely to be adequate as the mothers were consuming wheat flour products fortified with thiamine (2.5 mg/kg). Whether deficits in niacin existed is uncertain as the contribution of niacin from tryptophan was not included in the data presented in Table 4. In contrast, protein intakes exceeded the estimated needs from complementary foods at 9 and 12 months, although there was a slight shortfall at 6 months.

**Table 4:** Estimated need and median (IQR) energy and nutrient intakes from complementary food at 6, 9, and 12 months.

	6 months		9 months		12 months	
Nutrient	Need	Intake	Need	Intake	Need	Intake
* *	* *	*n* = 215	* *	*n* = 192	* *	*n* = 186
Energy (kcal)^a^	202	127 (95–166)	307	275 (218–378)	548	396 (302–492)
Energy (kcal/kg)^b^	177		251		340	
Protein (g)	4.4	3.7 (2.8–5.1)	5.6	7.9 (5.6–11.9)	6.9	12.4 (8.7–16.9)
Niacin (mg) ^c^	1.6	0.6 (0.3–1.0)	3.7	1.5 (0.9–1.9)	5.7	2.0 (1.3–2.7)
Riboflavin (mg)	0.09	0.09 (0.06–0.12)	0.22	0.15 (0.09–0.30)	0.33	0.26 (0.17–0.42)
Thiamin (mg)	0	0.08 (0.06–0.11)	0	0.14 (0.07–0.19)	0	0.18 (0.12–0.25)
Vitamin C (mg)	1	15 (10–21)	10	10 (4–20)	10	13 (5–22)
Vitamin A (*μ*g RAE)	44	77 (56–115)	75	53 (26–105)	87	57 (26–99)
Calcium (mg)	161	83 (55–116)	293	109 (69–144)	402	122 (85–170)
Iron (mg)^d^	9.1	3.1 (1.9–4.2)	9.1	2.4 (1.5–3.4)	5.6	2.7 (1.9–3.7)
Zinc (mg)^e^	2.5	1.2 (0.8–1.7)	2.6	1.5 (1.0–2.1)	1.7	2.0 (1.5–2.7)

^a,b^ FAO/WHO, 2004.

^c^ Does not include contribution of niacin from tryptophan.

^d^ Assuming medium bioavailability (10%) (WHO/FAO,2004).

^e^ Assuming mixed or refined vegetarian diets (Brown et al., 2004).

Nutrient densities (i.e., intakes per 100 kcal) were also examined relative to the desired nutrient densities. However, these results are not included here because the low energy intakes shown in Table 4 limit the interpretation of the nutrient densities.

### Intakes of energy and selected nutrients in relation to consumption of fortified infant foods

Significant differences existed for the median intakes of energy and several nutrients from complementary foods among infants at 9 months of age consuming fortified vs. non-fortified infant foods (Table 5). Of note is the significantly lower energy intake of infants consuming fortified foods, whereas intakes of vitamin C, vitamin A, calcium and iron (but not zinc) were higher (P<0.001), as expected. The MARs, summary indicators of the adequacy (as %) of all the nutrient intakes examined at 9 mo, differed according to whether infants were consuming (n = 69) or not consuming (n = 123) fortified infant foods (66±13% vs. 60±13%; P = 0.05), respectively at this time (data not shown).

**Table 5:** Median (IQR) of energy density, intakes of energy and nutrients of infants consuming and not consuming infant fortified foods at 9 months.

	9 months		
	Median (IQR)		p-value
	Fortified (n = 69)	Non-fortified (n = 123)	
Energy density (kcal/g)	1.2 (1.0–1.5)	1.5 (1.0–1.9)	0.006
Energy (kcal)	250 (206–293)	310 (237–400)	<0.001
Protein (g)	6.5 (5.2–8.7)	9.1 (6.2–12.7)	<0.001
Niacin (mg) ^a^	1.2 (0.8–1.8)	1.5 (0.9–2.0)	0.313
Riboflavin (mg)	0.14 (0.09–0.22)	0.16 (0.08–0.36)	0.493
Thiamine (mg)	0.14 (0.08–0.18)	0.14 (0.06–0.20)	0.815
Vitamin C (mg)	16 (9–27)	7 (3–14)	<0.001
Vitamin A (*μ*g RAE)	76 (50–144)	42 (16–84)	<0.001
Calcium (mg)	128 (87–168)	106 (53–133)	<0.001
Iron (mg)	2.7 (2.1–3.8)	2.2 (1.2–3.0)	<0.001
Zinc (mg)	1.4 (1.2–2.0)	1.6 (0.8–2.1)	0.692

^a^ Does not include contribution of niacin from tryptophan.

In general, no differences existed in the maternal and household status variables or the characteristics of those infants who consumed or did not consume fortified infant foods; notable exceptions were the maternal education and wealth index variables. Higher maternal education (p = 0.007) and wealth index score (p<0.001) was noted for those infants who consumed fortified infant foods compared to those who did not consume fortifed foods.

### Relationships between nutrient adequacy, complementary feeding indicators, sentinel food groups and subsequent growth

Data were not examined at 6 mo because so few infants met the MDD or MAD indicators (both 2.8%) (Table 3). At 9 mo, the NARs showed positive correlations with dietary diversity scores for all infants (r = 0.38; p<0.0001) and for those not consuming fortified infant foods (r = 0.54; p<0.0001), but not for those consuming fortified infant foods.

The relationship between three core WHO complementary feeding indicators at 9 months and subsequent growth at 12 months is shown in Table 6. Morbidity defined by diarrhea was not included in the model because it was not a significant variable in the univariate regression analysis. Of the three indicators, consumption of iron-rich/iron fortified infant foods at 9 months was the only indicator that was positively associated with linear growth (P = 0.04) at 12 months in the fully adjusted models. Of the four sentinel foods also investigated at 9 months, fortified infant foods alone were positively and significantly related to a greater LAZ and WAZ at 12 months. It is noteworthy that LAZ, WLZ, and WAZ had a stronger effect on subsequent growth at 12 months than minimum dietary diversity, with β-coefficients of ~0.8 for LAZ,~0.9 for WLZ; and ~ 1.0 for WAZ. No significant associations were observed with growth at 12 months for the average daily intakes at 9 months of the critical growth-limiting nutrients (protein, zinc, iron, calcium, and riboflavin) expressed per day or per kg body weight, or for the NARs for the infants (data not shown).

**Table 6:** WHO complementary feeding indicators and sentinel food indicators at aged 9 months in relation to LAZ, WLZ, and WAZ-scores at 12 months.

* *	Growth at 12 months		
Core WHO indicators at 9 mos	β	95% Conf. Interval	p-value
LAZ			
Minimum dietary diversity	0.01	-0.18, 0.20	0.91
Minimum acceptable diet	0.01	-0.18, 0.20	0.91
Iron-rich/iron fortified infant foods	0.22	0.01, 0.44	0.04
WLZ			
Minimum dietary diversity	0.05	-0.13, 0.24	0.58
Minimum acceptable diet	0.05	-0.13, 0.24	0.58
Iron-rich/iron fortified infant foods	-0.22	-0.42, 0.00	0.05
WAZ			
Minimum dietary diversity	-0.01	-0.13, 0.10	0.83
Minimum acceptable diet	-0.01	-0.13, 0.10	0.83
Iron-rich/iron fortified infant foods	-0.03	-0.15, 0.10	0.70
Sentinel food indicators at 9 mos			
LAZ			
Flesh foods	0.07	-0.12, 0.26	0.45
Eggs	0.02	-0.18, 0.21	0.88
Animal-source foods	0.10	-0.10, 0.30	0.32
Fortified infant foods	0.29	0.09, 0.48	0.04
WLZ			
Flesh foods	-0.11	-0.30, 0.07	0.24
Eggs	-0.05	-0.24, 0.14	0.59
Animal-source foods	-0.11	-0.31, 0.09	0.27
Fortified infant foods	-0.09	-0.29, 0.11	0.37
WAZ			
Flesh foods	-0.07	-0.18, 0.04	0.22
Eggs	-0.05	-0.16, 0.07	0.47
Animal-source foods	-0.06	-0.18, 0.06	0.32
Fortified infant foods	0.14	0.02, 0.26	0.02

Note: *Analysis was not conducted for dairy products as there were less than 15 observations in the category and all, except 2 children had minimum meal frequency. Model is adjusted for growth at 9 months (LAZ, WLZ, WAZ at 9 months), sex, mother’s height, wealth index (quintile), mother’s education, and mother’s height.

## Discussion

The increase in the prevalence of stunting from 6 to 12 months of age observed here was also reported in the 2010 Indonesian National Health Report [45] and has been seen in other low income countries in the region [46–48]. We found that transition from exclusive breastfeeding to complementary feeding in the Sumedang district was not in accordance with the WHO guiding principles for breastfed children [14], and the complementary diets were inadequate in both quantity and quality.

Unlike several earlier studies [49–51], we found no associations between either dietary diversity or MAD and subsequent growth. Instead, intake of iron-rich (i.e., flesh foods/iron fortified infant foods) at 9 months was associated with a greater LAZ at 12 months. When the effect of flesh foods and iron-fortified infant foods on growth was examined separately (Table 6), only the intake of fortified infant foods was associated with a greater LAZ (Table 6, β, 0.29, P = 0.04) and a greater WAZ (β, 0.14, P = 0.02) at 12 months. Lack of a positive effect of flesh foods on linear growth in this study is not unexpected given that major flesh food items consumed by the infants at aged 9 months were predominantly cereal-based meat balls and sausages.

The positive outcome of infant fortified foods on growth reported here in contrast to some earlier studies of micronutrient fortified complementary foods [52–56] may have arisen because most of the specialized infant foods consumed by almost all of the Sumedang infants at 6 months (91.7%) and about a third (35.8%) at 9 months were fortified, not only with micronutrients, but also with cow’s milk powder. The growth-stimulating effects of milk on growth are now recognized. Several investigators have shown that the consumption of cow’s milk, both unfortified [57] and fortified [58–61] promotes growth during childhood, perhaps through the stimulation of insulin growth factor-1 (IGF-1). Milk is also a source of several other growth-promoting constituents, including high quality protein, lactose, calcium, potassium, magnesium and phosphorus [62, 63].

Interestingly, the energy density the diets of those infants consuming fortified infant foods was lower than for the non-consumers (1.2 kcal/g vs. 1.5 kcal/g; P<0.006) at aged 9 months. Likewise, intakes of energy and protein (per day) of these consumers were significantly lower than for the non-consumers (Table 5). These findings probably arose because caregivers prepared porridges from these expensive fortified infant products with a lower dry matter content (e.g., diluted) in an effort to make them more long lasting. Hence, despite almost all infants achieving MMF, this practice may have been responsible, at least in part, for the deficits in some of the micronutrients observed when compared with the estimated needs (Table 4). Whether there were also shortfalls in energy is uncertain; estimates for absolute energy requirements shown in Table 4 could be over-estimated because of the small size of these infants together with their inability to experience full catch-up growth because of the challenges of overcoming inter-generational growth stunting [64, 65].

We did not calculate micronutrient density adequacies because the low energy intakes reported here distort the nutrient density data. Instead we calculated NARs and found a strong correlation between NARs and dietary diversity scores for the “non-consumers” of infant fortified foods (r = 0.54, P<0.0001), but not for consumers. Failure to observe a relationship between dietary diversity scores and NARs with the “consumers of fortified infant foods” has been reported elsewhere [44, 66], and is not unexpected as the single food group—fortified infant foods—supplied ~ 56%, 50%, and 49%, of the total intake of iron, zinc, and calcium, respectively at 9 months. These findings highlight that even in this underprivileged rural setting, consumption of expensive fortified infant foods limits the usefulness of dietary diversity scores as a proxy for micronutrient adequacy. However, MDD is intended as an overall measure of dietary quality, not only indicating micronutrient adequacy, but also a high likelihood of consuming ≥ 1 animal-source food and ≥ 1 fruit or vegetable in addition to a staple food [41]. Further, infants benefit from increased dietary diversity as the exposure to novel foods of differing textures and flavors facilitates the development of healthy food preferences [67].

### Strengths and limitations

We employed a longitudinal design, and collected a comprehensive range of qualitative measures and quantitative nutrient intakes at the individual level making it possible to identify potential dietary causes of impaired growth among infants who reside in Sumedang district. Nevertheless, we restricted our evaluation of nutrient adequacy to eight key micronutrients, and excluded vitamin B-6, vitamin B-12, and folate included elsewhere [44, 69] because of the paucity of reliable Indonesian nutrient composition values for these micronutrients.

We also acknowledge that despite collecting two-day in-home weighed food intakes, our data on energy and nutrient adequacies has some uncertainties. For our population we assumed literature values for breast milk volume and composition rather than direct measurements, even though poor maternal status has the potential to reduce breast milk concentrations of vitamin A, riboflavin and niacin [38], all micronutrients seemingly “at risk” in the complementary diets reported here. Finally, we chose to use the WHO/FAO RNIs, except for zinc rather than the most recent values compiled by EFSA [70] to facilitate comparison of our data with published reports in other low income countries. It is also important to note that the consistently lower proportion of infants at each age meeting the MDD and MAD and consuming the four sentinel food groups and fortified infant foods from day 1 only instead of the two non-consecutive days collected here highlights the importance of collecting data representative of usual intakes, whenever possible, to avoid misleading conclusions [68].

### Implications for programs

Our results indicate that despite receiving fortified infant foods, intakes of selected micronutrients from complementary foods for Sumedang infants across all three ages failed to meet the estimated needs. Caregivers should prepare infant fortified foods with the recommended dry matter content. However, they should not be encouraged to over-rely on fortified infant foods as they reduce dietary diversity. Instead, caregivers should be encouraged to increase the consumption of appropriate and affordable animal-source foods (i.e., dairy products, flesh foods, and eggs) and fruits and vegetables over the complementary feeding period to ensure the infant’s diets achieve the WHO indicators for MDD and MAD. In this way infants will become familiar with a variety of textures and flavors, and will consume potentially important bioactive compounds not present in fortified infant foods [67, 71]. Finally, our results emphasize the use of a local sentinel food group**─**fortified infant foods**─**as a practical indicator in this setting for predicting subsequent infant growth.

## Supporting information

S1 Dataset Infant and young child feeding practices of infants in Sumedang district, Indonesia. (XLSX)

S1 Table Number and percentage of infants meeting five core WHO complementary feeding indicators and consuming four sentinel food groups at 6, 9, and 12 months on day 1. (PDF)
