# Supplementary material for: Consumption of fortified infant foods reduces dietary diversity but has a positive effect on subsequent growth in infants from Sumedang district, Indonesia
Source: PLoS One. 2017 Apr 20;12(4):e0175952. doi: 10.1371/journal.pone.0175952 (PMC5398566; doi:10.1371/journal.pone.0175952)
Supplement: S1 Table — (PDF) [file pone.0175952.s002.pdf]

S1 Table. Number and percentage of infants meeting five core WHO complementary feeding indicators and consuming four sentinel food groups at 6, 9, and 12 months on day 1

WHO IYCF Indicator	6 mo<br>n=218	9 mo<br>n=193	12 mo<br>n=186
Introduction of solid, semi-solid, or soft foods	217 (99.5)	NA	NA
Minimum dietary diversity (≥ 4 food groups)	3 (1.4)	44 (22.8)	70 (37.6)
Minimum meal frequency	190 (87.2)	180 (93.3)	182 (97.8)
Minimum acceptable diet	3 (1.4)	44 (22.8)	69 (37.1)
Consumption of iron-rich/iron fortified infant foods	193 (88.5)	117 (60.6)	117 (62.9)
Sentinel food groups			
Dairy products	8 (3.7)	3 (1.6)	6 (3.2)
Flesh foods	2 (0.9)	74 (38.3)	104 (55.9)
Eggs	1 (0.5)	40 (20.7)	59 (31.7)
Animal-source foods	11 (5.0)	97 (50.3)	133 (71.5)
Fortified infant foods	192 (88.1)	55 (28.5)	18 (9.7)
